# Characteristics of evidence-based medicine training in Royal College of Physicians and Surgeons of Canada emergency medicine residencies - a national survey of program directors

**DOI:** 10.1186/1472-6920-14-57

**Published:** 2014-03-21

**Authors:** Joseph Bednarczyk, Merril Pauls, Jason Fridfinnson, Erin Weldon

**Affiliations:** 1 Department of Emergency Medicine, University of Manitoba, Old Basic Medical Sciences Bldg, T258F-770 Bannatyne Avenue, Winnipeg, Manitoba R3E 0W3, Canada

**Keywords:** Evidence-based medicine, Medical education, Emergency medicine, E-learning, Journal club

## Abstract

**Background:**

Recent surveys suggest few emergency medicine (EM) training programs have formal evidence-based medicine (EBM) or journal club curricula. Our primary objective was to describe the methods of EBM training in Royal College of Physicians and Surgeons of Canada (RCPSC) EM residencies. Secondary objectives were to explore attitudes regarding current educational practices including e-learning, investigate barriers to journal club and EBM education, and assess the desire for national collaboration.

**Methods:**

A 16-question survey containing binary, open-ended, and 5-pt Likert scale questions was distributed to the 14 RCPSC-EM program directors. Proportions of respondents (%), median, and IQR are reported.

**Results:**

The response rate was 93% (13/14). Most programs (85%) had established EBM curricula. Curricula content was delivered most frequently via journal club, with 62% of programs having 10 or more sessions annually. Less than half of journal clubs (46%) were led consistently by EBM experts. Four programs did not use a critical appraisal tool in their sessions (31%). Additional teaching formats included didactic and small group sessions, self-directed e-learning, EBM workshops, and library tutorials. 54% of programs operated educational websites with EBM resources. Program directors attributed highest importance to two core goals in EBM training curricula: critical appraisal of medical literature, and application of literature to patient care (85% rating 5 - “most importance”, respectively). Podcasts, blogs, and online journal clubs were valued for EBM teaching roles including creating exposure to literature (4, IQR 1.5) and linking literature to clinical practice experience (4, IQR 1.5) (1-no merit, 5-strong merit). Five of thirteen respondents rated lack of expert leadership and trained faculty educators as potential limitations to EBM education. The majority of respondents supported the creation of a national unified EBM educational resource (4, IQR 1) (1-no support, 5- strongly support).

**Conclusions:**

RCPSC-EM programs have established EBM teaching curricula and deliver content most frequently via journal club. A lack of EBM expert educators may limit content delivery at certain sites. Program directors supported the nationalization of EBM educational resources. A growing usage of electronic resources may represent an avenue to link national EBM educational expertise, facilitating future collaborative educational efforts.

## Background

The Royal College of Physicians and Surgeons of Canada (RCPSC) provides a five-year training program in Emergency Medicine (EM) with special emphasis on developing academic emergency physicians [1]. The goal of training for fellows of the RCPSC is to prepare learners for roles in university centers. Academic emergency positions require expertise in medical education, research, and administration [2]. Graduates are expected to apply evidence-based medicine (EBM) in scholarly and managerial roles [3] (Table 1). Practically, the structured approach of EBM has particular importance in the emergency department, with high levels of diagnostic uncertainty, decision density, and time pressure [4,5].

**Table 1:** Royal College of Physicians and Surgeons of Canada emergency medicine training requirements in evidence-based medicine

**CanMeds role**	**Requirements for completion of training**
**Scholar**	Access and interpret the relevant evidence
	Critically appraise evidence to address a clinical question
	Integrate critical appraisal conclusions into clinical care
**Manager**	Apply best available medical evidence and management for cost-appropriate care
	Plan relevant emergency department operations based upon evidence gathered through the use of information technology

EBM education in EM remains in the early stages of development. A recent survey of the Council of Emergency Medicine Residency Directors in the United States (US) indicates a minority of programs had predefined journal club or EBM curricula (25%) or used structured critical appraisal instruments in their journal clubs (29%) [6]. Kuhn and colleagues reported only 22% of EM program directors in the US conducted more than five didactic EBM sessions per year [7]. In both studies, program directors expressed a desire for a defined curriculum [6,7]. Such work has established a baseline in teaching methods and outlined a needs assessment for training programs in the US, however this first step has not been performed in Canada.

In EM residencies, EBM content delivery has been described and formats include traditional journal club, structured “evidence-based” journal club, didactic formats, and web based EM blogs [8-11]. Specific concerns have been raised regarding the narrow focus placed on critical appraisal during journal club sessions, however the optimal educational method has not been defined [12,13]. Adding complexity, the rapid expansion of web based educational technology has changed the way learners access, analyze, and catalogue new evidence [14].

Before evaluating EBM training in EM, current practices must be defined. Our primary objective was to describe the methods of EBM training in RCPSC-EM residencies. Secondary objectives were to (i) explore attitudes regarding current educational practices, including e-learning, (ii) explore potential barriers to journal club and EBM education, and (iii) assess the desire for national collaboration. By defining educational methods across institutions and identifying areas for growth, we hope to create a platform for local and national quality improvement initiatives relevant to EBM education in EM.

## Methods

### Study design and setting

A paper-based survey of all RCPSC-EM program directors in Canada was performed. Ethics approval was obtained from the University of Manitoba Health Research Ethics Board. Contact information for programs was obtained from the Canadian Resident Matching Service (CaRMS) website [15]. A cover letter stated survey completion was adequate evidence of informed consent. Respondents received a fifty-dollar honoraria directed to their resident education fund.

### Survey content and administration

An anonymous 16-question survey containing binary, open ended, and 5 point likert-scale questions was developed by the authors. Survey content was refined by consensus discussion during local focus group meetings and informal collaboration with national EBM experts in EM. We explored the following domains: (i) EBM curricula format and core values, (ii) journal club characteristics and limitations, (iii) use and perceived merit of electronic/web based EBM resources, and (iv) basic demographics. All likert scales provided opportunity for neutral responses. Several questions offered “other” selections and free text to elicit alternate responses.

Program directors were mailed introductions to the research initiative beginning in July 2012. Surveys were mailed one week later with a cover letter indicating estimated completion time, voluntariness, honoraria value, and confidentiality via aggregate data reporting. Potential respondents were provided postage paid return envelopes addressed to an administrative assistant who removed identifying packaging before returning data to the authors. Non-respondents received two bimonthly email requests to complete the survey. Responses were collected until August 2012.

### Data analysis

Data were stored using Numbers ’09 (Apple Inc. Version 2.1 (c) 2008-2011). Descriptive statistical analysis was performed. Because of the obligatory small sample size and descriptive nature of the study objective, formal sample size calculations and statistical significance were not performed. To preserve the integrity of data trends in likert scale questions, results are represented in both median and percentage where applicable.

## Results

**Figure 1:**
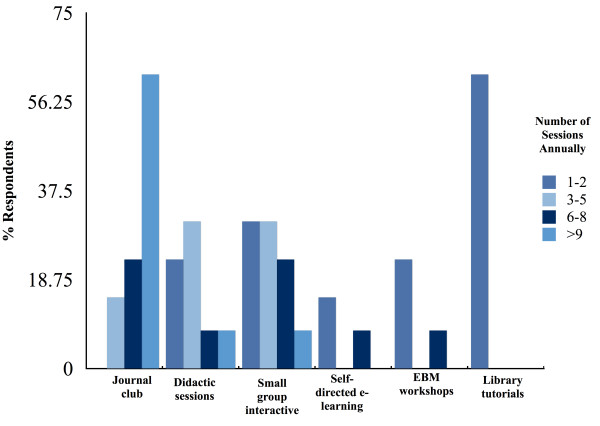
Frequency of teaching modalities in EBM curricula.

Fourteen RCPSC-EM programs were identified, of which 13 programs (93%) responded. Seven (54%) respondent programs belonged to EM programs with departmental status. Additional demographics of respondents are represented in (Table 2). Eleven respondent programs (85%) reported having formal EBM teaching curricula. Ten programs (77%) had a lead EBM educational coordinator. Curricula content was delivered primarily via journal club, with 62% of programs having 10 or more sessions annually. Teaching formats utilized less frequently included didactic and small group sessions, self-directed e-learning, EBM workshops, and library tutorials (Figure 1).

**Table 2:** Demographics of respondents

**Age of FRCP residency program**	**1-5 y**	**6-10 y**	**11-15 y**	**16-20 y**	**>20 y**
n (%)	1 (8)	1 (8)	2 (15)	3 (23)	6 (46)
**Number of residents in program**	**1-11**	**12-21**	**23-31**	**32-41**	**>41**
n (%)	2 (15)	5 (38)	2 (15)	2 (15)	2 (15)

Article selection for journal club occurred most frequently via resident or attending choice (85% and 54% respectively). Four programs (31%) had predefined journal club curricula, and only one program reported journal club article selection from clinical questions generated on shift. Six programs (46%) indicated journal club sessions were consistently led by an EBM expert, defined as an individual with formal training in EBM, extensive background in clinical research, or a longstanding academic interest in biostatistics and research methodology. Nine programs (69%) used a critical appraisal tool in their journal club sessions. Respondents who specified their critical appraisal tool used the JAMA User’s Guide (5/9) or Oxford critical appraisal worksheets (1/9) [16,17]. Sessions were held at staff residences or university sites in most cases (38% and 48% respectively), with only 2 programs (15%) holding journal club at public venues.

Noise, alcohol, and distractions in public venues were thought to compromise the educational value of journal club, with several programs reporting some (6 (46%)) or significant (2 (15%)) limitation. Five respondents perceived lack of expert leadership and trained faculty educators as posing some (3,23%) or significant (2,15%) limitation. “Narrow focus on critical appraisal” was not frequently rated as a significant limitation to journal club (Table 3).

**Table 3:** Perceived limitations of journal club: n (%) respondents

	**No limitation 1**	**2**	**Neutral 3**	**4**	**Significant limitation 5**
**Public venue (noise, distractions)**	4(31)	0	1(8)	6(46)	2(15)
**Narrow focus on critical appraisal**	3(23)	1(8)	5(38)	3(23)	1(8)
**Lack of expert leadership**	3(23)	1(8)	4(31)	3(23)	2(15)

**Figure 2:**
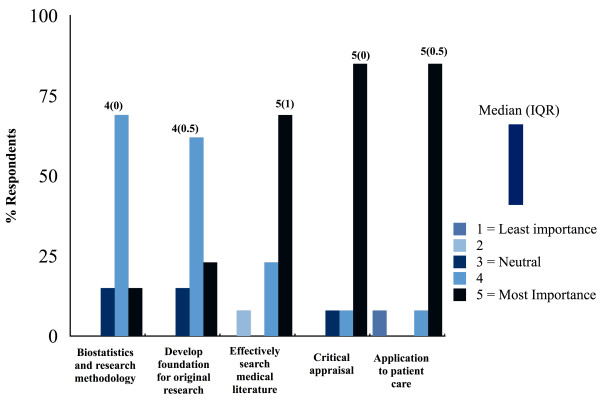
Core values in EBM curricula.

Program directors attributed highest importance to two core goals in EBM training curricula: critical appraisal of medical literature, and application of literature to patient care (85% rating 5 -“most importance”, respectively). Effective search strategy was also highly valued, with 69% of respondents rating “most importance” to this objective. Conversely, understanding biostatistics and developing a foundation for producing original research were less frequently attributed “most importance” in EBM curricula (15 and 23% respectively) (Figure 2).

**Figure 3:**
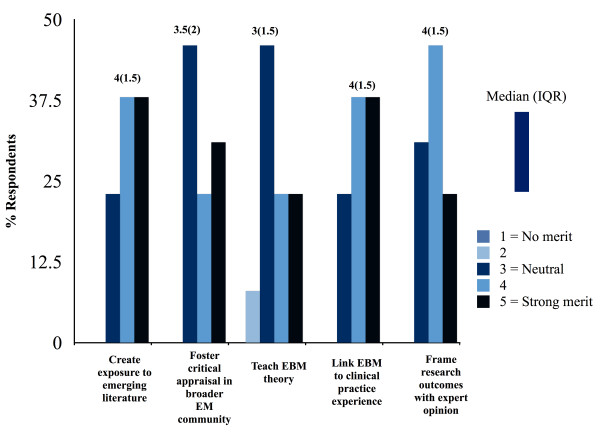
Perceived merit of electronic resources in EBM education.

Over half of programs operated educational websites with links to external EBM resources and documented journal club summaries electronically (7 (54%) respectively). E-resources including podcasts, blogs, and online journal clubs were valued for certain roles as components of EBM education (Figure 3). Most respondents supported the role of e-resources in creating exposure to emerging literature and linking EBM to clinical practice experience ((5, 38%) rating “4”, (5 (38%) rating “5”, 1 -no merit, 5 - strong merit). However, program directors were more likely to report a neutral response relating to the merit of e-resources in teaching specific aspects of EBM theory (6 (46%) rating “3”-neutral). Respondents indicated referring residents to e-resources that are not formally peer-reviewed may be hazardous (median response “4”, IQR 1; 1-insignificant danger, 5-significant danger).

Only four programs (31%) had evaluated the effectiveness of their EBM teaching methods or journal club in a quality improvement initiative in the last three years. Despite this, several respondents indicated limited EBM trained faculty educators may pose a barrier to effective EBM teaching (Table 4). 46% of respondents strongly supported the creation of a national unified EBM educational resource for the purposes of making EBM expert educators available, linking journal clubs, and creating a sustainable critically appraised topic (C.A.T) database (median 4, IQR1, 1-no support, 5 – strongly support).

**Table 4:** Potential barriers to effective EBM teaching

	**Insignificant barrier 1**	**2**	**Neutral 3**	**4**	**Significant barrier 5**
**Limited EBM trained faculty educators**	2(15)	1(8)	5 (38)	4(31)	1(8)
**Lack of funding for EBM training**	1(8)	3(23)	6(46)	3(23)	0
**Lack of interest in department**	3(23)	3(23)	5(38)	2(15)	0

## Discussion

To our knowledge, this is the first effort to describe the landscape of EBM training in Canadian EM residencies. Compared with previous reports of EM training programs in the US, EBM teaching curricula were prominent in nearly all survey respondents. While the aim of this study was not to thoroughly review individual curricula, several observations may serve as a basis for self-evaluation and quality improvement initiatives.

The EBM skill set enables an EM trainee to make evidenced-based patient care decisions, but begins with the ability to seek out and appraise resources based on a resident’s clinical questions [12,18]. A successful training program teaches each step of the EBM process, producing physicians who incorporate EBM as a behavior in their daily practice [17]. Although respondents attributed some importance to each component of EBM, critical appraisal was highly valued. This was also evidenced by the predominance of journal club - a critical appraisal exercise - as the most frequently reported educational activity among EBM curricula.

Much research has focused on journal club as an EBM teaching tool in EM [8-10,19]. The efficacy of this traditional format to teach the breadth of EBM skills or change behavior has been questioned [10,11,20]. In a systematic review of journal club in post-graduate medical education, none of the 101 articles retrieved demonstrated translation of evidence from journal club into the clinical practice of learners [21]. Because of a narrow focus on critical appraisal, journal club may be an unsatisfactory method of teaching the full scope of evidence based decision making [22]. Furthermore, as Green argues: “critical appraisal may be the most expendable skill for the ‘front line’ physician practicing evidence-based medicine in the ‘real world’” [22]. Despite these growing concerns, few respondents in our survey identified the narrow focus on critical appraisal as a significant limitation to their journal clubs or EBM curricula.

Recommendations from an extensive review on improving journal club effectiveness included regular meetings, mandatory attendance, and use of a critical appraisal tool [21]. These respective criteria were reported by 92, 100, and 69% of respondents in our survey. Yet perhaps the most consistent suggestion in EBM education literature is to engage learners in the breadth of the EBM process with applied techniques, beginning and ending at the patient’s bedside [11,23,24]. Curricula incorporating experiential learning may best meet the needs of adult learners [22,23,25]. As demonstrated in an observational cohort, Friedman et al. described a knowledge translation intervention consisting of a 1 hour tutorial and real time EBM exercise in the emergency department [23]. This encouraging work showed a change in patient management in 16.3% of encounters and the potential to impact future practice in resident learners. Of concern, only one of our respondent programs indicated residents generated journal club topics from clinical questions arising on shift. This may represent an important area for growth in Canadian EM curricula.

Additional teaching formats were used with lesser frequency among respondents - including didactic sessions and library tutorials. Such formats are supported by the literature, as Grant demonstrated a didactic intervention was effective in teaching EBM theory to EM residents [26]. Of much interest, however, is the use and support of e- resources by EM programs in resident education, with over half of programs operating EBM websites and cataloging critically appraised topics. Web 2.0, a term encompassing the collaborative use of information technology (blogs, social media, podcasts) has increasing relevance to EM education [14]. With over twelve thousand MEDLINE articles added weekly, EBM curricula must also educate residents to identify sources of pre-appraised evidence [27,28]. Peer reviewed sources such as Academic Emergency Medicine, Annals of Emergency Medicine, and Emergency Medicine Journal are among the contributors to over 200 EM blogs (catalogued at http://lifeinthefastlane.com) which may help learners filter this vast amount of information [29-32]. Given the inherent variability in quality of such resources, however, PD’s may wish to develop and incorporate methods of critical appraisal of web-based secondary information sources into resident education.

Our survey demonstrated less than half of programs had consistent leadership by EBM experts at journal club sessions. In addition to being accessible, current, and asynchronous, web-based EBM resources also create a non-hierarchal environment where junior learners can interact with experts in the field, and participate in critical appraisal on a broader scale [14,33]. Linking respective journal clubs or online EBM resources between institutions may create a bridge for smaller centers with fewer EBM educators to larger institutions with surplus leadership and research experience. Based on the demographic data in this survey, respondents varied widely in program maturity and size. The authors speculate that the five respondents that cited lack of EBM expert leadership and limited faculty educators as limitations to EBM training may belong to younger, smaller programs. Indeed, this further supports the case for linking our nations EBM resources, a proposition met with great support by survey respondents. We feel this may represent an exciting opportunity to transform and strengthen EBM training in Canadian EM, by uniting institutions on a web-based EBM educational platform.

## Limitations

Our survey instrument was not prospectively tested for validity. Respondents represented the lead education coordinators for RCPSC-EM residents, but their opinions may differ from those of respective institutional EBM leaders. Furthermore, because of the relative paucity of specialty EM training programs in Canada, our sample size was small. This may have contributed to type I or II error. Future study defining the values and needs of residents in EBM education may be valuable. Ongoing efforts to identify educational interventions that change behavior in learners have great importance for EBM curricula.

## Conclusions

The majority of RCPSC-EM programs have prioritized EBM education by developing dedicated curricula. EBM training is diverse, however journal club and critical appraisal appear to be emphasized. This may not optimize the accomplishment of all core EBM training goals valued by respondents. If continued at the reported frequency, journal club sessions designed for resident education should incorporate applied, patient centered methods based on principles of adult learning theory, increase the use of critical appraisal tools, and assure EBM experts are involved in sessions. Lack of EBM leadership at some centers may hinder optimal resident education. A growing usage and support of web-based resources may represent an avenue to link EBM educators across Canada, and respond to the desire expressed by program directors to create a national unified EBM educational resource.

## Abbreviations

RCPSC: Royal College of Physicians and Surgeons of Canada; EM: Emergency medicine; US: United States; EBM: Evidence-based medicine; CaRMS: Canadian resident matching service; E-learning: Learning and teaching formats involving electronic media.

## Competing interests

The authors have no financial or other conflicts of interest to disclose.

## Authors’ contributions

JB contributed to project conception and design, data acquisition, data analysis and interpretation, and manuscript composition. MP contributed to project conception and design, revision of survey instrument and manuscript revision/critical appraisal. JF contributed to project conception and design, design of survey instrument, data acquisition and manuscript revision. EW contributed to overall inception and project supervision, project conception and design, data analysis and interpretation, and senior manuscript revisions. All authors give final approval for publication and are accountable for all aspects of the work’s accuracy and integrity.

## Pre-publication history

The pre-publication history for this paper can be accessed here:

http://www.biomedcentral.com/1472-6920/14/57/prepub
